# Prognostic value of a circadian rhythm-related gene signature in breast cancer patients: A retrospective cohort study

**DOI:** 10.1097/MD.0000000000043882

**Published:** 2025-08-15

**Authors:** Tielin Wang, Ying Liu, Yan Zhang, Hong Liu, Jiechao Ren, Jian Wu

**Affiliations:** a Center of Breast and Thyroid Surgery, Department of General Surgery, Chengdu The Third People’s Hospital of Chengdu, Affiliated Hospital of Southwest Jiaotong University & The Second Affiliated Hospital of Chengdu, Chongqing Medical University, Chengdu, China; b Department of Ultrasound, The Third People’s Hospital of Chengdu, Affiliated Hospital of Southwest Jiaotong University & The Second Affiliated Hospital of Chengdu, Chongqing Medical University, Chengdu, China.

**Keywords:** bioinformatics, breast cancer, circadian rhythm-related genes, prognostic signature, survival prediction

## Abstract

By 2020, breast cancer (BRCA) surpassed lung cancer as the most prevalent cancer globally, exhibiting high morbidity and mortality. Given the emerging role of circadian rhythm in cancer progression, this study aimed to develop a prognostic signature based on circadian rhythm-related genes (CRRGs) to predict BRCA patient survival. Gene expression profiles and clinical data were sourced from the Gene Expression Omnibus (GEO), The Cancer Genome Atlas (TCGA), and the Molecular Signatures Database (MSigDB). A multigene signature was constructed using LASSO-penalized Cox regression. Patients were stratified into high- and low-risk groups based on median risk scores. Pathway activity was assessed via gene set variation analysis (GSVA). Prognostic performance was evaluated using ROC curves, Kaplan–Meier (K-M) analysis, and multivariate Cox regression. A 20-CRRG prognostic signature was identified, effectively stratifying patients into distinct risk groups (K-M *P* < .05). ROC analysis demonstrated high predictive accuracy (AUC > 0.7). Functional enrichment linked these CRRGs to circadian regulation, nuclear components, and DNA binding. Further refinement revealed a 9-gene subset (ADRB1, BHLHE41, BTG1, EGR3, NONO, NR1H3, NTRK3, OPN4, PIGF) with superior 5-year survival prediction (AUC 0.82) compared to 1- and 3-year outcomes. The CRRG-based signature, particularly the 9-gene subset, robustly predicts BRCA patient survival, offering potential clinical utility for long-term prognosis. These findings underscore the role of circadian rhythms in BRCA progression and highlight novel biomarkers for risk stratification.

## 1. Introduction

Breast cancer (BRCA) had exceeded lung cancer by 2020, claiming its position as the most prevalent cancer globally, exhibiting high morbidity and mortality.^[1]^ According to the latest research and data, the field of breast cancer (BRCA) treatment has made significant progress in the past few decades, especially in endocrine therapy, targeted therapy, and chemotherapy. However, breast cancer remains one of the leading causes of cancer-related deaths among women worldwide. In 2020, there were about 2.26 million to 2.3 million new cases of breast cancer and about 685 000 deaths worldwide.^[2–4]^ Some studies have reported that the clock genes exert an impact on tumorigenesis. The regulation of gene expression and cell cycle is governed by clock genes, which play a direct role in controlling cell division, cell proliferation, apoptosis, cell cycle checkpoints, and response to DNA damage.^[5–7]^ They could increase the risk of cancer development when these functions are altered. The exploration of molecular alterations in circadian rhythm-regulated genes within breast tumor cells remains largely uncharted. Therefore, identifying the molecular mechanisms underlying BRCA development is essential for advancing cancer therapy. Clock genes, constituting an auto-regulated transcription-translational loop of circadian genes, have a vital part in driving the circadian rhythm within individual cells, thereby fulfilling vital physiological necessities.^[8]^ In certain tissues (i.e. breast tumors, endometrial carcinoma, and lung cancer), circadian proteins undergo alterations in tumor cells in contrast to their adjacent normal cells. Some clock genes might act as oncogenes, while others might act as tumor-inhibiting factors.^[9,10]^ However, whether the prognosis of BRCA patients is associated with circadian rhythm-related genes (CRRGs) remains unclear. Now that humans have entered the era of genes and big data, we could explore the relationship between BRCA and circadian rhythm at the genetic, and molecular levels through sequencing combined with bioinformatics.

In this study, first, we collected BRCA samples data, gene expression profiles as well as their corresponding clinical data of BRCA patients from the databases of Gene Expression Omnibus (GEO), UCSC Xena, and The Cancer Genome Atlas (TCGA). And we utilized Molecular Signatures Database (MSigDB) to acquire comprehensive information about the human gene set. Subsequently we selected 282 CRRGs from a literature of the PubMed website by organizing, merging, and eliminating the weight. The prognostic multigene signature incorporating differentially expressed genes (DEGs) associated with circadian rhythm was founded. The predictive efficacy of prognostic signature was determined. Also, the credibility was verified by performing GEO database. Finally, we further explored association between BRCA and molecular functions (MF), biological processes (BP), as well as cellular components (CC) by conducting functional enrichment analysis (FEA).

## 2. Methods

### 2.1. Ethical statement

This study utilized publicly available de-identified genomic and clinical data from TCGA and GEO databases. As all data were obtained from open-access repositories with preexisting institutional review board approval, no additional ethical approval was required for this secondary analysis. The original studies from which these data were derived received appropriate ethical oversight from their respective institutions. Since the study involved retrospective analysis of anonymized data without any direct patient interaction or intervention, the requirement for informed consent was waived. All methods were performed in accordance with relevant guidelines and regulations for bioinformatics research.

### 2.2. Data collection

Utilizing “TCGAbiolinks” R package, data on gene expression (counts) and corresponding clinical information were acquired for 1109 BRCA and 113 para-carcinoma samples from TCGA up to October 01, 2022 (https://portal.gdc.cancer.gov/).^[11]^ Then, 28 samples with incomplete clinical data were eliminated. Thus, we included 1083 BRCA samples (TCGA-BRCA) and 111 para-carcinoma samples with integrate follow-up data into the training data set for subsequent analyses. We standardized the data to Fragments per Kilobaseper Million (FPKM) data, and acquired relevant clinical data utilizing UCSC Xena database (http://genome.ucsc.edu). The count sequencing data for the data set (TCGA-BRCA) were standardized by adopting “limma”R package.^[12]^

Besides, we implemented “GEOquery” R package and acquired another BRCA-related data set adopting GEO (https://www.ncbi.nlm.nih.gov/geo/).^[13]^ Based upon GPL570 (Affymetrix Human Genome U133 Plus 2.0 Array), GSE42568^[14]^ was conducted, encompassing microarray gene-expression profiles of 104 BRCA samples and 17 normal control (non-tumor samples). The microarray gene-expression profiles of 683 BRCA samples were analyzed using the GPL570 platform (Affymetrix Human Genome U133 Plus 2.0 Array) in the study GSE102484.^[15]^ All the samples were encompassed in our research. Data set probe names were annotated using the chip GPL platform file.

MSigDB^[16]^ provides comprehensive information about human gene sets. We searched a literature for circadian rhythm-related gene set,^[17]^ and obtained a total of 300 CRRGs after weight removal. Then we removed the missing genes missing during the probe transformation of the data set and obtained a total of 282 CRRGs.

### 2.3. Construction of a prognostic model

To obtain a prognostic model for CRRGs in BRCA, we used a 10-fold cross-validation and implemented regression adopting least absolute shrinkage and selection operator (LASSO) method with seed number 2021 and *P*-value = .05, and ran 1000 cycles for each cycle to prevent overfitting. After visualizing the results of LASSO regression, we proceeded to present an illustration of the sample grouping, survival outcome, as well as the molecular expression of prognostic CRRGs of each group. The risk factor graph consists of 3 parts: Risk grouping, based upon median risk score that obtained from LASSO regression prognostic model prediction for all samples of TCGA-BRCA data set, the disease group was allocated to low-risk group (LRG) and high-risk group (HRG). Survival outcome, dot plots were used to demonstrate the survival time and outcomes of the clinical samples in TCGA-BRCA data set. Heatmap, the expression levels of prognostic CRRGs in LASSO regression prognostic model were visualized in the model samples.

### 2.4. Analysis of DEGs in both groups

To screen the underlying mechanism of action and related biological characteristics as well as pathways in HRG and LRG of the BRCA samples, first, the BRCA dataset GSE42568, GSE102484 was used to remove the combined GEO data set (BRCA dataset), and subsequently, the dataset was subjected to a comparative analysis prior to and following the debatch effect through the distribution boxplot and principal component analysis (PCA) plot. Next, “limma” R package was adopted to pair TCGA-BRCA dataset and BRCA dataset for standardized and high and low-risk grouping processing. That is, after excluding the normal group of samples, the median risk score bound of the LASSO model constructed by prognostic CRRGs was allocated to BRCA HRG (group: High) and BRCA LRG (group: Low) and the differential analysis of the processed expression profile data. DEGs between different groups of 2 BRCA datasets were identified, with *P* < .05 and logFC > 0 as DEGs with up-regulation, and *P* < .05 and logFC < 0 as DEGs with downregulation. The “ggplot2” R package and “pheatmap” R package were adopted to draw volcano plots and heat maps and display the difference analysis results. To examine the hub gene localization in pairs of chromosomes, UCSC database (http://genome.ucsc.edu/) was employed to first analyze the start and stop sequence of hub genes. Afterward, the chromosome location mapping was drawn adopting the “RCircos” R package.^[18]^ The “GOSemSim” R package^[19]^ was employed to estimate the functional correlation of key genes, and the functional correlation between prognostic CRRGs was analyzed.

### 2.5. Analysis of gene set function and enrichment and variation

We performed GO annotation analysis of prognostic CRRGs employing the “clusterProfiler” R package.^[20]^ The screening standards were FDR value (q.value) < 0.2 and P. adj < 0.05, indicating significant difference, with Benjamini-Hochberg implemented for *P*-value correction.

Gene set enrichment analysis (GSEA)^[21]^ was applied to judge the contribution of genes from pre-defined gene sets to the phenotype. In this study, the genes in the dataset TCGA-BRCA were allocated to 2 groups of phenotype-related risk based upon the median ranking of Risk Score obtained by LASSO. Then, we implemented enrichment analysis for all genes in the 2 groups of phenotype-associated risk adopting R package clusterProfiler. For GSEA, the following parameters were set: 2020 as seed, 1000 as number of calculation times, 10 and 500 as the least and the maximum number of genes of each gene set, respectively. *P*-value correction was implemented employing Benjamini-Hochberg method. MSigDB was employed to acquire the c2.cp.v7.2, with FDR (q.value) < 0.25 and *P* < .05 as the screen criterion for enrichment significance.

For the sake of assessing whether there was enrichment of distinct pathways among the various samples, we obtained “ h.all.v7 in the MSigDB database. 4.symbols.gmt “ Gene sets performed the GSVA of the dataset TCGA-BRCA separately at the levels of gene expressions to compute the difference of functional enrichment between the 2 tissues.

### 2.6. Prognostic analysis

We plotted subgroup K-M curves for circadian-related prognostic DEGs in the LASSO model against subtype clinical variables, with *P* = .05 as the threshold to search for associated genes with statistically significant differences.

### 2.7. Protein–protein interaction (PPI)

PPI involves the interaction of individual proteins with one another. We utilized prognostic CRRGs screened out from STRING database (https://cn.string-db.org/, Access: August 2022)^[22]^ constructed PPI networks associated with DEGs [low confidence (0.150) in option of least required interaction score]. Local regions closely interconnected within PPI network might correspond to molecular complexes exhibiting distinct biological functions. Cytoscape software^[23]^(v3.9.1) was adopted for visualization of PPI network model. The functional similar genes of the selected prognostic CRRGs were predicted employing GeneMANIA Website^[24]^ and the interaction work was established.

### 2.8. ROC curve

ROC curve^[25]^ of prognostic CRRGs in the dataset TCGA-BRCA was plotted utilizing survival ROC package of R. Area under curve (AUC) was computed to assess the diagnostic efficacy of prognostic CRRGs expression on the survival of BRCA patients. Then we included the genes with an AUC > 0.6 in prognostic CRRGs as the key genes. Immunohistochemical (IHC) analysis was implemented on the expression of key genes identified through Cox models utilizing the Human Protein Atlas database (www.proteinatlas.org/).

### 2.9. Correlation analysis of the prognosis

For purpose of exploring the prognostic value of key genes for BRCA, the hub gene expression in TCGA-BRCA data set was examined, and univariate Cox regression for clinical variables was also implemented. Factors meeting *P < .1* were chosen for incorporation into the multivariate Cox regression analysis, thus forming a robust and comprehensive multivariate Cox regression model. Based upon the multivariate Cox regression analysis results, a nomogram was created for the prediction of the 1-year, 3-year, and 5-year survivals of BRCA patients. We drew DCA plots with R package ggDCA^[26]^ to assess the predictive efficacy of nomogram model for the survival outcomes at 1 year, 3 years, and 5 years of BRCA patients.

### 2.10. Statistical methods

R software (v4.1.2) was executed to complete the entire data processing and analyses. The continuous variables were displayed as mean ± standard deviation. Wilcoxon rank sum test was implemented to compare continuous variables between groups. Independent Student *t* test and the Kruskal-Wallis test were adopted to estimate the significant difference of variables with normal distribution. Chi-square tests or Fisher exact tests were utilized to analyze the statistical significance of categorical variables between 2 groups. If not specifically indicated, spearman correlation analysis was implemented for result calculations between different molecules, and all P statistics were 2-sided, with *P* < .05 as the standard for statistical significance.

## 3. Results

### 3.1. Construction of the prognostic model as well as the differential expression analysis

**Figure 1.**
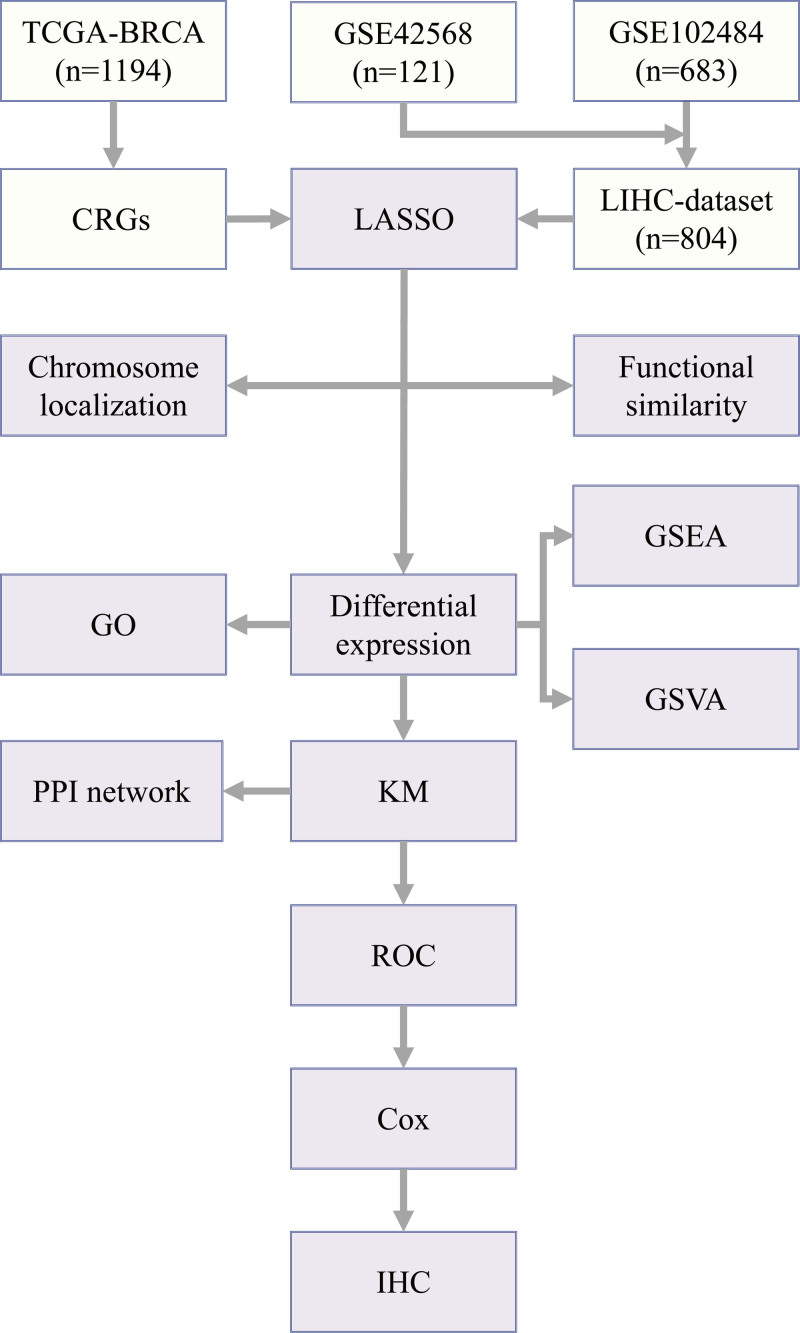
Overview of the research process. BRCA = breast cancer, CRRGs = circadian rhythm-related genes, GO = gene ontology, GSEA = gene set enrichment analysis, GSVA = gene set variation analysis, IHC = immunohistochemical analysis, K-M = Kaplan–Meier curve, LASSO = least absolute shrinkage and selection operator, PPI = protein–protein interaction, ROC = receiver operating characteristic curve, TCGA = The Cancer Genome Atlas.

**Figure 2.**
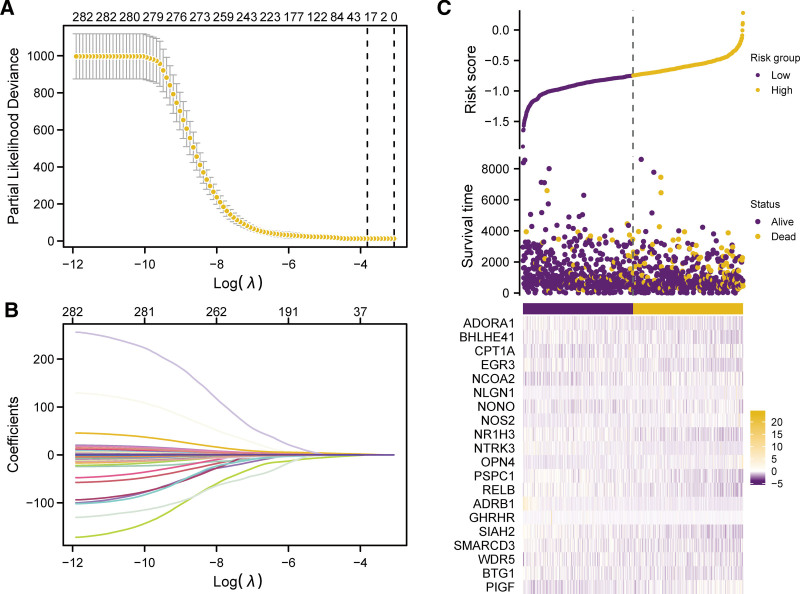
CRRGs construction of the prognostic models. Diagnostic model plot of the LASSO regression for CRRGs. (B–C) Graph of variable trajectory (B), risk factor graph of LASSO regression prognostic model (C). The ordinate of prognostic model diagram (A) indicates the likelihood deviation value, and in the lower part of the figure, the x-axis displays the logarithm (log) of λ, indicating the log after lambda coefficient of the penalty term in LASSO regression; in the upper part of the figure, the x-axis represents variable number with the corresponding coefficient non-zero under each lambda. The yellow dots in the risk factor plot (C) represent the deceased patients, and the purple dots represent the surviving patients. CRRGs = circadian rhythm-related genes, LASSO = least absolute shrinkage and selection operator.

Our workflow was depicted in Figure 1. With the aim of determining the prognostic value of 282 CRRGs (detailed in Table S1, Supplemental Digital Content, https://links.lww.com/MD/P675) in dataset TCGA-BRCA, LASSO regression analysis was executed to build a prognostic model (Fig. 2A), with 20 CRRGs in the model (ADORA1, ADRB1, BHLHE41, BTG1, CPT1A, EGR3, GHRHR, NCOA2, NLGN1, NONO, NOS2, NR1H3, NTRK3, OPN4, PIGF, PSPC1, RELB, SIAH2, SMARCD3, WDR5) (Table 1). The LASSO regression outcomes were visualized, and the graph representing the trajectory of LASSO variables were generated (Fig. 2B). Then, in the established prognostic model of LASSO regression analysis, the risk factor graph was applied for visualizing the sample grouping situation (Fig. 2C). From the scatter plot of the risk factor chart, the population with low-risk displayed slightly longer survival time compared with the population with high risk.

**Table 1:** List of gene symbol of circadian rhythm prognosis-related genes.

Gene symbol				
ADORA1	ADRB1	BHLHE41	BTG1	CPT1A
EGR3	GHRHR	NCOA2	NLGN1	NONO
NOS2	NR1H3	NTRK3	OPN4	PIGF
PSPC1	RELB	SIAH2	SMARCD3	WDR5

### 3.2. Analysis of DEGs in HRG and LRG of BRCA patients

**Figure 3.**
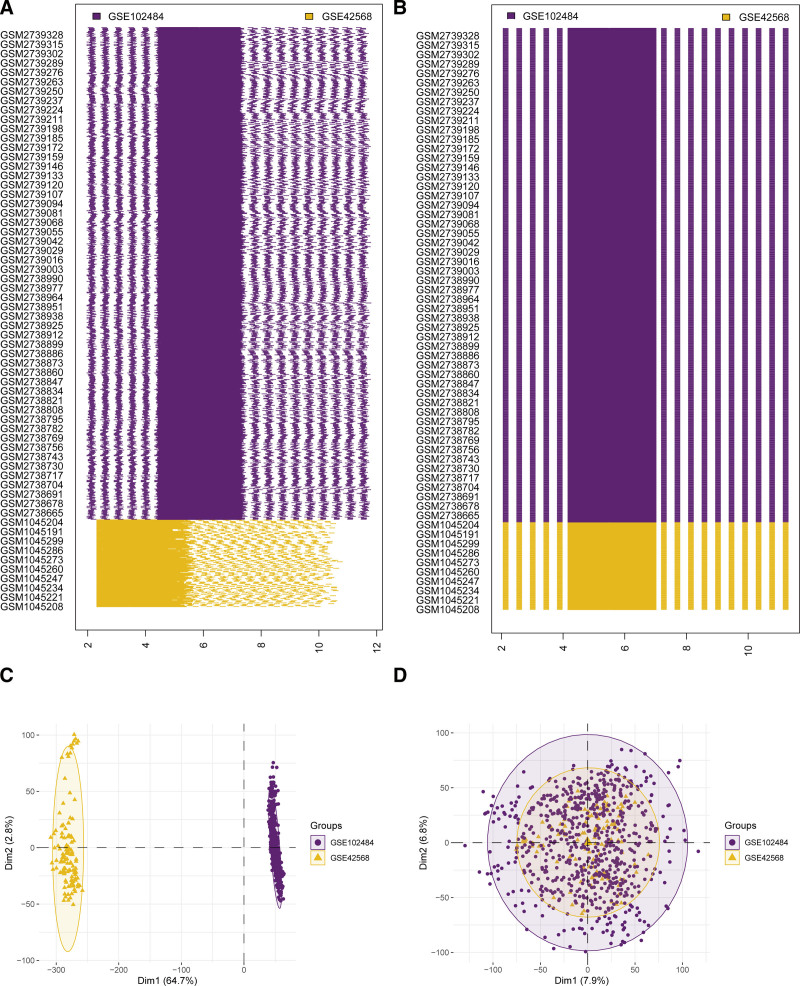
Boxplot and PCA plot of sample distribution before and after BRCA dataset incorporation. (A–B) Boxplot of the sample distribution before (A) and after (B) for the incorporation of the dataset BRCA dataset. (C–D) PCA plot of pre- (C) post- (D) merge of dataset BRCA dataset. BRCA = breast cancer, PCA= principal component analysis.

**Figure 4.**
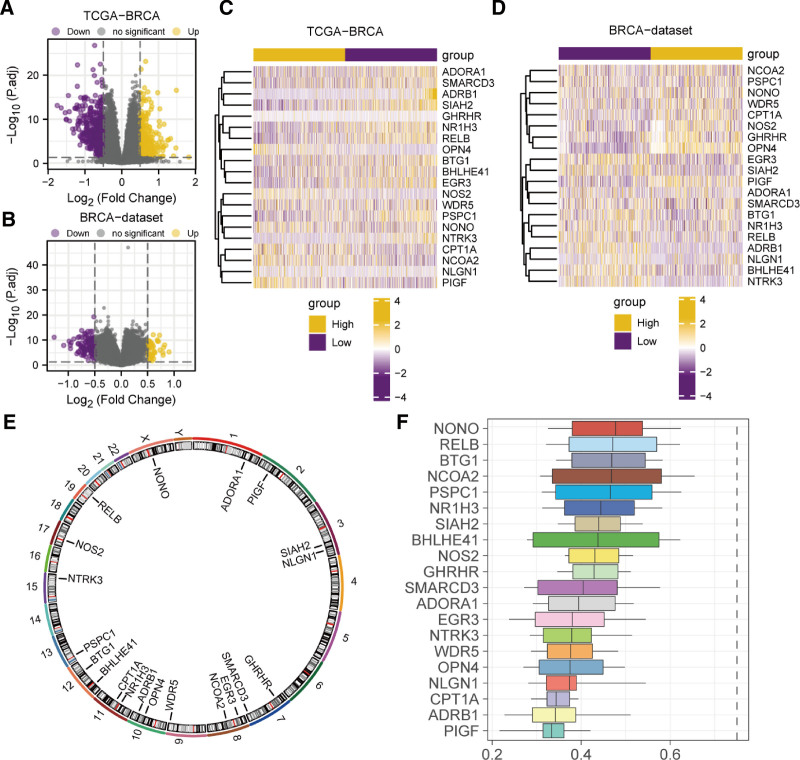
Analysis of differential genes between HRG and LRG of BRCA. (B) Volcano plots of DEGs between HRG (group: High) and LRG (group: Low) of BRCA in TCGA-BRCA (A), BRCA dataset (B). (C–D) Complex numerical heatmap of genes involved in circadian prognosis in dataset TCGA-BRCA (C), BRCA dataset (D). (E) Chromosome mapping map of CRRGs. (F) Bar chart depiction of the functional similarity analysis outcomes of CRRGs. BRCA = breast cancer, CRRGs = circadian rhythm-related genes, DEGs = differentially expressed genes, HRG = high-risk group, LRG = low-risk group, TCGA = The Cancer Genome Atlas.

Based upon the findings of the abovementioned plots, after undergoing batch removal treatment, the BRCA dataset experienced large elimination in batch effect (Fig. 3A–D). We acquired 19,426 DEGs in total in the data set TCGA-BRCA, including 844 genes fulfilling | logFC |> 0.5 and *P. adj < .05*. At such threshold, 325 genes had high expression in breast risk group (low expression, upregulated genes, positive logFC), and 519 genes had low expression in breast risk group (high expression in LRG, negative logFC). A volcano plot was generated to present the differential analysis outcomes of this data set (Fig. 4A). We acquired 21,655 DEGs in total in BRCA dataset, including 149 genes fulfilling | logFC |> 0.5 and *P. adj < 0.05*. At such threshold, 43 positive logFC (genes with up-regulation) and 106 negative logFC (genes with downregulation) were acquired. The differential analysis outcomes of this data set were displayed utilizing a volcano plot (Fig. 4B). We analyzed 20 CRRGs (ADORA1, ADRB1, BHLHE41, BTG1, CPT1A, EGR3, GHRHR, NCOA2, NLGN1, NONO, NOS2, NR1H3, NTRK3, OPN4, PIGF, PSPC1, RELB, SIAH2, SMARCD3, WDR 5) (Table 2) to know the differential expression between HRG and LRG in datasets TCGA-BRCA (Fig. 4E) and BRCA (Fig. 4F). Heat map was depicted employing R package pheatmap (Fig. 4E–F).

**Table 2:** List of description and expression difference of circadian rhythm prognosis-related genes.

Gene symbol	Description	LogFC	*P*-value	adj.*P*
ADORA1	Adenosine a1 receptor	−0.606548629	3.61713E−09	5.59892E−08
ADRB1	Adrenoceptor beta 1	−1.206610634	3.05746E−21	3.12601E−18
BHLHE41	Basic Helix-Loop-Helix Family Member E41	−0.415251909	1.28284E−08	1.68154E−07
BTG1	BTG Anti-Proliferation Factor 1	−0.270365304	1.61613E−09	2.82076E−08
CPT1A	Carnitine Palmitoyltransferase 1A	0.321981779	1.53981E−08	1.98622E−07
EGR3	Early Growth Response 3	−0.925903726	8.15693E−18	2.17064E−15
GHRHR	Growth Hormone Releasing Hormone Receptor	−0.268814815	.016778235	.036449788
NCOA2	Nuclear Receptor Coactivator 2	0.37584189	1.41789E−14	1.18214E−12
NLGN1	Neuroligin 1	0.073618565	.540659119	.63650332
NONO	Non-POU Domain Containing Octamer Binding	0.037049573	.134253715	.210103332
NOS2	Nitric Oxide Synthase 2	0.503058216	1.93879E−13	1.14826E−11
NR1H3	Nuclear Receptor Subfamily 1 Group H Member 3	−0.384535793	3.15796E−18	1.02244E−15
NTRK3	Neurotrophic Receptor Tyrosine Kinase 3	−1.116671797	3.81206E−18	1.13928E−15
OPN4	Opsin 4	0.498490446	2.6999E−12	1.09495E−10
PIGF	Phosphatidylinositol Glycan Anchor Biosynthesis Class F	0.296017624	1.85272E−22	3.2256E−19
PSPC1	Paraspeckle Component 1	−0.202219907	1.49543E−13	9.25169E−12
RELB	RELB Proto-Oncogene, NF-KB Subunit	−0.580459286	4.39275E−27	2.84445E−23
SIAH2	Siah E3 Ubiquitin Protein Ligase 2	−0.736484758	7.75903E−32	1.50727E−27
SMARCD3	SWI/SNF Related, Matrix Associated, Actin Dependent Regulator Of Chromatin, Subfamily D, Member 3	−0.29228627	2.09863E−06	1.47048E−05
WDR5	WD Repeat Domain 5	−0.030336962	.283562301	.385693969

To analyze the positions of the 20 CRRGs on the human chromosome, we also annotated these 20 CRRGs with positions using the R package RCircos (Fig. 4E). As shown in the graph, the ADORA1 gene is located on chromosome 1. The PIGF gene is located on chromosome 2. The SIAH2 and NLGN1 gene are located on chromosome 3. The GHRHR and SMARCD3 gene are located on chromosome 7. The EGR3 and NCOA2 gene are located on chromosome 8. The WDR5 gene is located on chromosome 9. The OPN4 and ADRB1 gene are located on chromosome 10. The NR1H3 and CPT1A are located on chromosome 11. The BHLHE41and BTG1 gene are located on chromosome 12. The PSPC1 gene is located on chromosome 13. The NTRK3 gene is located on chromosome 15. The NOS 2 gene is located on chromosome 17. The RELB gene is located on chromosome 19. The NONO gene is located on the X chromosome. The CRRGs distributed at the same chromosomal locations are more closely linked at the genomic level. Functional similarity analysis evaluates the functional similarity of genes to other genes and shown as bar graphs according to the level of the scores. The figure shows that the gene NONO has the highest functional similarity score with the other genes.

### 3.3. Gene ontology

**Figure 5.**
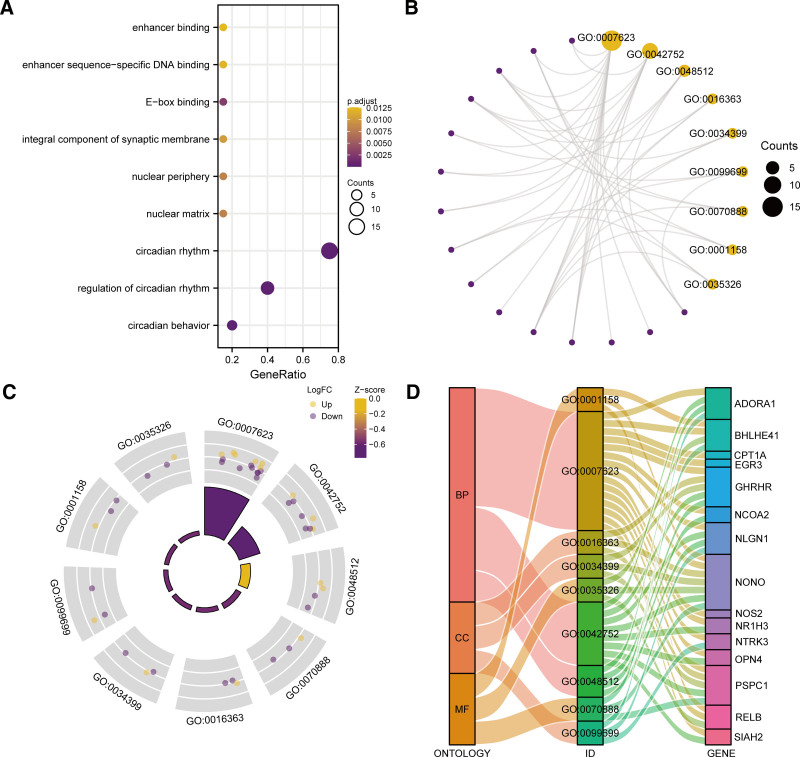
FEA (GO). (A–B) Bubble mapping (A) and circular network graph (B) presentation of GO enrichment analysis outcomes of prognostic CRRGs. (C) Circle plots presentation of GO enrichment outcomes of combined logFC of prognostic CRRGs. In network graph (B), purple dots and yellow circles indicate specific genes and pathways, respectively. In the circle plot (C), yellow and purple dots signify up- (logFC > 0) and down-regulated (logFC < 0) genes, respectively. FDR (q.value) < 0.2 and *P* < .05 were deemed to screening standards for GO enrichment entries. CRRGs = circadian rhythm-related genes, FEA = functional enrichment analysis, GO = gene ontology.

In order to analyze 20 genes (ADORA1, ADRB1, BHLHE41, BTG1, CPT1A, EGR3, GHRHR, NCOA2, NLGN1, NONO, NOS2, NR1H3, NTRK3, OPN4, PIGF, PSPC1, RELB, SIAH2, SMARCD3, WDR5), and their BP, MF, CC and BRCA. We performed GO gene FEA for these 20 circadian rhythm prognosis-related genes (Table 3). The screening criteria for enriched items were *P < .05* and FDR value (*q*.value) < 0.25. The results showed that these 20 circadian rhythm prognosis-related genes were mainly enriched in the BRCA genes of circadian rhythm, regulation of circadian rhythm, In biological process (BP) such as circadian behavior, and nuclear matrix, nuclear periphery, cellular component (CC) such as integral component of synaptic membrane, and E-box binding, enhancer sequence-specific DNA binding, enhancer binding and other MF. The results of GO enrichment analysis are presented in bubble plots (Fig. 5A). We also present the results of GO enrichment analysis in the form of a network diagram (Fig. 5B). We then performed a combined logFC GO enrichment analysis on these 20 circadian rhythm prognosis-related genes, that is, based on the enrichment analysis, The logFC value of the provided molecules (20 circadian rhythm prognosis-related genes) in the results of the difference analysis of the BRCA data set TCGA-BRCA high and LRGs was used to calculate the corresponding z score. We show the GO enrichment analysis results of joint logFC by circle diagram (Fig. 5C), and show the GO enrichment analysis results by Sankey diagram (Fig. 5D) in the form of categories (ONTOLOGY, including BP, CC, and Ontology). MF) and the relationship between the corresponding function or pathway number (ID) and the GENE name (GENE) included in the category number (ID). Circle diagram showed that circadian rhythm (ID, GO:0007623) and regulation of circadian rhythm (ID, GO:0042752) were significantly upregulated BP.

**Table 3:** GO enrichment analysis results of circadian rhythm prognosis-related genes.

Ontology	ID	Description	GeneRatio	BgRatio	*P*-value	*P*.adjust	qvalue
BP	GO:0007623	Circadian rhythm	15/20	208/18,670	4.48E−26	4.94E−23	2.99E−23
BP	GO:0042752	Regulation of circadian rhythm	8/20	114/18,670	1.78E−13	6.56E−11	3.97E−11
BP	GO:0048512	Circadian behavior	4/20	46/18,670	1.52E−07	3.66E−05	2.21E−05
CC	GO:0016363	Nuclear matrix	3/20	109/19,717	1.75E−04	.008	0.005
CC	GO:0034399	Nuclear periphery	3/20	131/19,717	3.01E−04	.008	0.005
CC	GO:0099699	Integral component of synaptic membrane	3/20	152/19,717	4.65E−04	.010	0.007
MF	GO:0070888	E-box binding	3/20	50/17,697	2.34E−05	.003	0.002
MF	GO:0001158	Enhancer sequence-specific DNA binding	3/20	119/17,697	3.11E−04	.012	0.008
MF	GO:0035326	Enhancer binding	3/20	133/17,697	4.31E−04	.013	0.008

### 3.4. Gene set enrichment analysis

**Figure 6.**
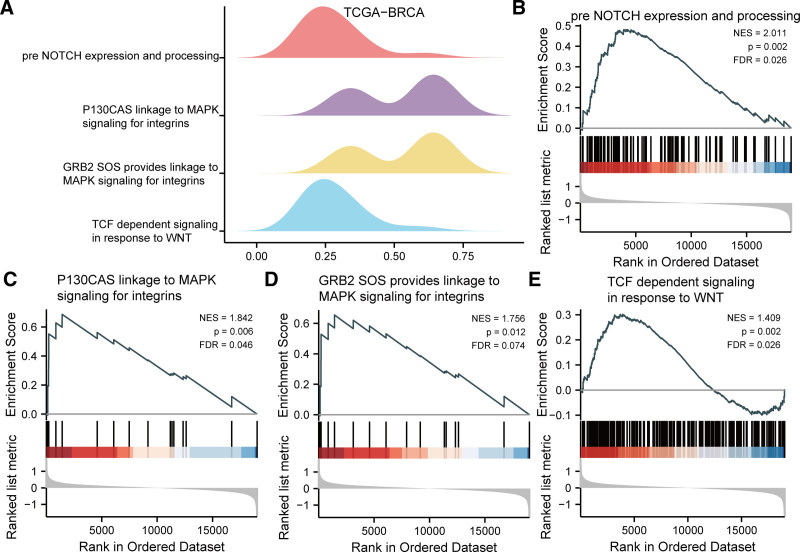
GSEA. (A) Four primary biological characteristics of GSEA in data set TCGA-BRCA. (B–E) In the data set TCGA-BRCA, genes were significantly enriched in pre NOTCH expression and processing (B), P130CAS linkage to MAPK signaling for integrins (C), GRB2_SOS_provides_linkage_to_MAPK_signaling_for_integrins (D), TCF dependent signaling in response to WNT (E), etc. The screen standard for enrichment significance in GSEA was set at *P* < .05, FDR (q.value) < 0.25. BRCA = breast cancer, GSEA = gene set enrichment analysis, TCGA = The Cancer Genome Atlas.

With the aim of determining the impact of gene expression levels on differences between HRG and LRG of patients with BRCA, the expression of the entire genes and involved BP, CC affected, and the connection between exerted MF were analyzed by GSEA with *P* < .05 and FDR (q.value) < 0.25. Based upon the outcomes, genes were significantly enriched in pre NOTCH expression and processing in TCGA-BRCA (Fig. 6B), P130CAS linkage to MAPK signaling for integrins (Fig. 6C), GRB2_SOS_provides_linkage_to_MAPK_signaling_for_integrins (Fig. 6D), TCF dependent signaling in response to WNT (Fig. 6E), etc (Table 4). We presented the mountain map (Fig. 6A) and the pathway map (Fig. 6B–E) in the dataset TCGA-BRCA.

**Table 4:** GSEA analysis of dataset TCGA-BRCA.

Description	setSize	Enrichmentscore	NES	*P*-value	*P*.adjust
DNA methylation	63	0.680807247	2.601274469	.002123142	.031325174
Condensation of prophase chromosomes	72	0.661552425	2.580353795	.002207506	.031325174
SIRT1 negatively regulates RRNA expression	66	0.651290195	2.513278271	.002173913	.031325174
Olfactory signaling pathway	372	0.508648681	2.499726093	.002604167	.032740685
Meiotic recombination	85	0.624292764	2.490898128	.002272727	.031325174
Activated PKN1 stimulates transcription of AR androgen receptor regulated genes KLK2 and KLK3	65	0.648826127	2.488246889	.002169197	.031325174
PRC2 methylates histones and DNA	71	0.63781732	2.472704927	.002232143	.031325174
Olfactory transduction	368	0.498210243	2.442238242	.002638522	.032912096
Deposition of new cenpa containing nucleosomes at the centromere	73	0.618782939	2.422120642	.002207506	.031325174
Starch and sucrose metabolism	51	0.653065124	2.378526224	.002150538	.031325174
Porphyrin and chlorophyl metabolism	41	0.679813236	2.363575291	.002079002	.031325174
Pre NOTCH expression and processing	107	0.481539558	2.010542743	.002277904	.031325174
P130CAS linkage to MAPK signaling for integrins	15	0.686534175	1.841780552	.006198347	.056069018
GRB2 SOS provides linkage to MAPK signaling for integrins	15	0.654624365	1.756175393	.012396694	.090679522
TCF dependent signaling in response to WNT	230	0.301605068	1.40856886	0.002386635	0.031325174

BRCA = breast cancer, GSEA = gene set enrichment analysis, TCGA = The Cancer Genome Atlas.

### 3.5. Gene set variation analysis

**Figure 7.**
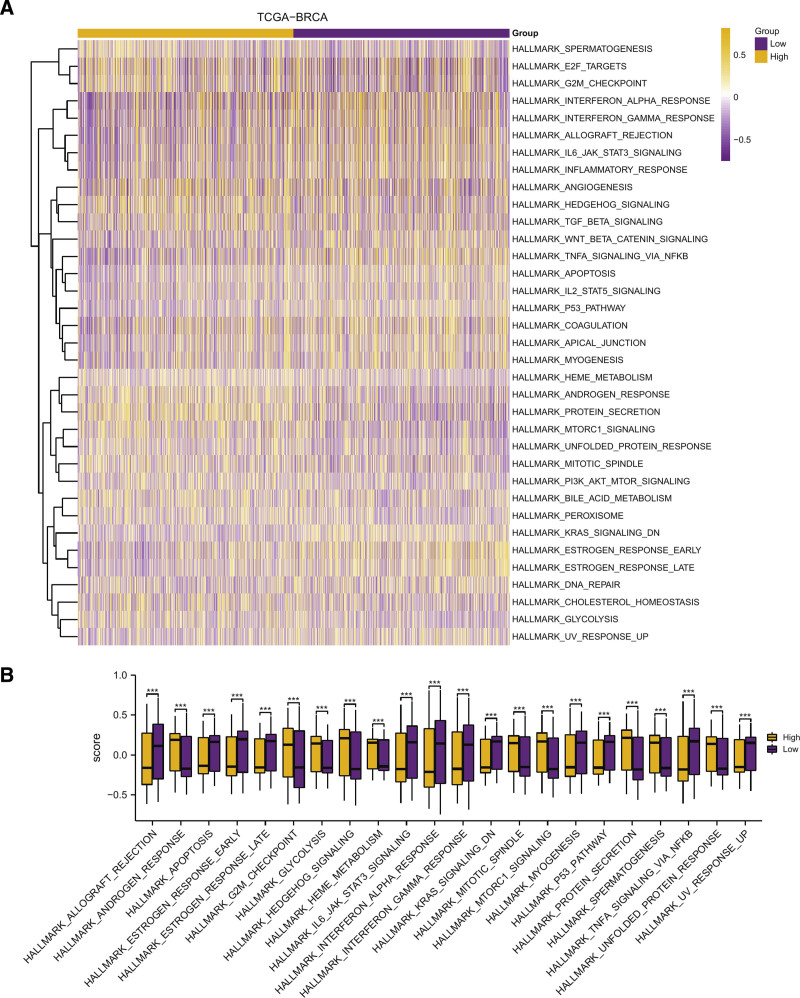
GSVA. Heatmap presentation of functional scores in the GSVA by dataset TCGA-BRCA. (B) Group comparison diagram of the enriched pathways that are very statistically significant between HRG and LRG of BRCA in the data set TCGA-BRCA. The horizontal axis is the sample, the vertical axis is the biological function, the node color signifies the inhibition or activation of the corresponding function, purple indicates inhibition, and yellow indicates activation. *** Represents *P*-value < 0.001, highly statistically significant. BRCA = breast cancer, GSVA = gene set variation analysis, HRG = high-risk group, LRG = low-risk group, TCGA = The Cancer Genome Atlas.

For the sake of examining in differences of hallmark gene set between HRG and LRG of BRCA, a GSVA was subsequently executed on entire gene expressions in TCGA-BRCA dataset. In GSVA of TCGA-BRCA, 35 hallmark gene sets including HALLMARK ALLOGRAFT REJECTION and HALLMARK ANDROGEN RESPONSE showed differences between 2 groups (Fig. 7A, Table 5). We selected the hallmark gene set with a statistically significant difference (*P < .001*) to draw a group comparison map (Fig. 7B). In the GSVA of TCGA-BRCA, 22 hallmark gene sets were statistically significant between HRG and LRG (*P < .001*) (Fig. 7B).

**Table 5:** GSVA analysis of dataset TCGA-BRCA.

Ontology	LogFC	AveExpr	*t*	*P*-value	adj.*P*
HALLMARK_PROTEIN_SECRETION	−0.16050262	0.016014509	−9.218257226	1.52E−19	7.62E−18
HALLMARK_KRAS_SIGNALING_DN	0.093528778	0.011189756	7.117016893	2.00E−12	5.00E−11
HALLMARK_TNFA_SIGNALING_VIA_NFKB	0.129124793	0.005953862	6.660681554	4.32E−11	7.20E−10
HALLMARK_ANDROGEN_RESPONSE	−0.101740714	0.014857923	−6.344031064	3.28E−10	4.10E−09
HALLMARK_MTORC1_SIGNALING	−0.105005335	−0.009756148	−6.226907624	6.79E−10	5.71E−09
HALLMARK_P53_PATHWAY	0.087192723	0.00193136	6.225390119	6.85E−10	5.71E−09
HALLMARK_ESTROGEN_RESPONSE_LATE	0.089962551	0.014295645	6.036708811	2.16E−09	1.54E−08
HALLMARK_ESTROGEN_RESPONSE_EARLY	0.09967299	0.026987255	5.929401871	4.08E−09	2.55E−08
HALLMARK_GLYCOLYSIS	−0.065293975	−0.013756156	−4.564654184	5.57E−06	3.10E−05
HALLMARK_INTERFERON_ALPHA_RESPONSE	0.10974441	−0.012308714	4.338444744	1.57E−05	7.84E−05
HALLMARK_HEDGEHOG_SIGNALING	−0.080499772	0.021088342	−4.100825366	4.42E−05	.000201096
HALLMARK_ALLOGRAFT_REJECTION	0.091750556	−0.006581609	4.053900408	5.40E−05	.000205976
HALLMARK_IL6_JAK_STAT3_SIGNALING	0.085574298	0.003091926	4.051470031	5.45E−05	.000205976
HALLMARK_SPERMATOGENESIS	−0.063732725	−0.009786995	−4.038101201	5.77E−05	.000205976
HALLMARK_MITOTIC_SPINDLE	−0.06377624	−0.002882936	−4.007962343	6.54E−05	.000218087
HALLMARK_HEME_METABOLISM	−0.048496498	0.011700639	−3.956432535	8.10E−05	.000253176
HALLMARK_UNFOLDED_PROTEIN_RESPONSE	−0.055581569	−0.012244708	−3.786413598	.000161211	.00047415
HALLMARK_APOPTOSIS	0.056260974	0.013916653	3.754804081	.00018268	.000507445
HALLMARK_G2M_CHECKPOINT	−0.080067263	−0.01822822	−3.655526225	.000268943	.000707744
HALLMARK_INTERFERON_GAMMA_RESPONSE	0.084366585	−0.006600445	3.638470918	.00028716	.0007179
HALLMARK_UV_RESPONSE_UP	0.048371985	−8.24E−05	3.54246102	.00041325	.000983929
HALLMARK_MYOGENESIS	0.060226781	0.007964877	3.470668748	.00053957	.001226296
HALLMARK_INFLAMMATORY_RESPONSE	0.064311326	0.004563335	3.197668919	.001425472	.003098853
HALLMARK_WNT_BETA_CATENIN_SIGNALING	0.05274809	0.00103605	2.976399069	.002981201	.006210836
HALLMARK_PEROXISOME	−0.038713628	0.004200688	−2.850882408	.004442358	.008884715
HALLMARK_APICAL_JUNCTION	0.045255061	0.013532118	2.703309174	.0069725	.013408653
HALLMARK_DNA_REPAIR	0.041119145	−0.002245849	2.688706252	.007282805	.013486677
HALLMARK_E2F_TARGETS	−0.063744625	−0.018993578	−2.655401401	.008037409	.014352517
HALLMARK_BILE_ACID_METABOLISM	−0.038429331	0.017005723	−2.549345605	.010929143	.018843349
HALLMARK_CHOLESTEROL_HOMEOSTASIS	−0.039081459	−0.003782115	−2.416076738	.015852984	.026421641
HALLMARK_IL2_STAT5_SIGNALING	0.036590802	0.001215229	2.332213645	.019871576	.032050929
HALLMARK_PI3K_AKT_MTOR_SIGNALING	−0.031489975	0.002450041	−2.273750901	.023175574	.036211835
HALLMARK_TGF_BETA_SIGNALING	−0.040909888	0.012228072	−2.132853981	.033160546	.050243251
HALLMARK_COAGULATION	0.040468574	0.021076445	2.066154214	.039050722	.057427533
HALLMARK_ANGIOGENESIS	−0.045848045	0.019515665	−2.003172063	.045407064	.064867234

BRCA = breast cancer, GSVA = gene set variation analysis, TCGA = The Cancer Genome Atlas.

### 3.6. Prognostic analysis and PPI networks

**Figure 8.**
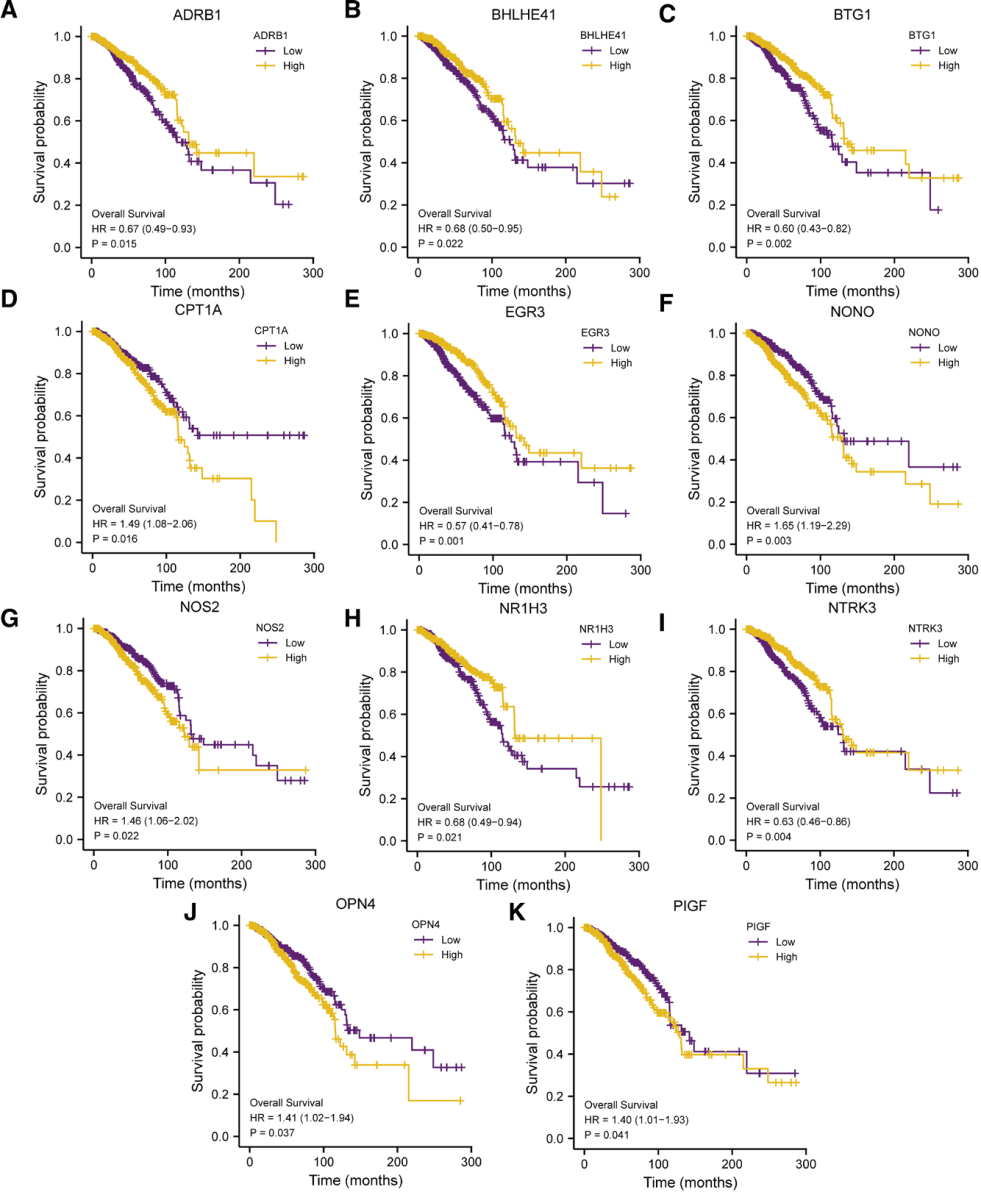
K-M curves. (A–K) K-M curve for the prognosis analysis of genes. ADRB1 (A), BHLHE41 (B), BTG1 (C), CPT1A (D), EGR3 (E), NONO (F), NOS2 (G), NR1H3 (H), NTRK3 (I), OPN4 (J), PIGF (K). K-M = Kaplan–Meier.

Eleven eligible genes were significant. Among them, the HR of CPT1A and NONO was > 1, and patients with high-risk genotypes had a higher risk of death (Fig. 8A–K). Eleven eligible genes (ADRB1, BHLHE41, BTG1, CPT1A, EGR3, NONO, NOS2, NR1H3, NTRK3, OPN4, PIGF) were obtained when significant difference was determined at *P < .05.*

**Figure 9.**
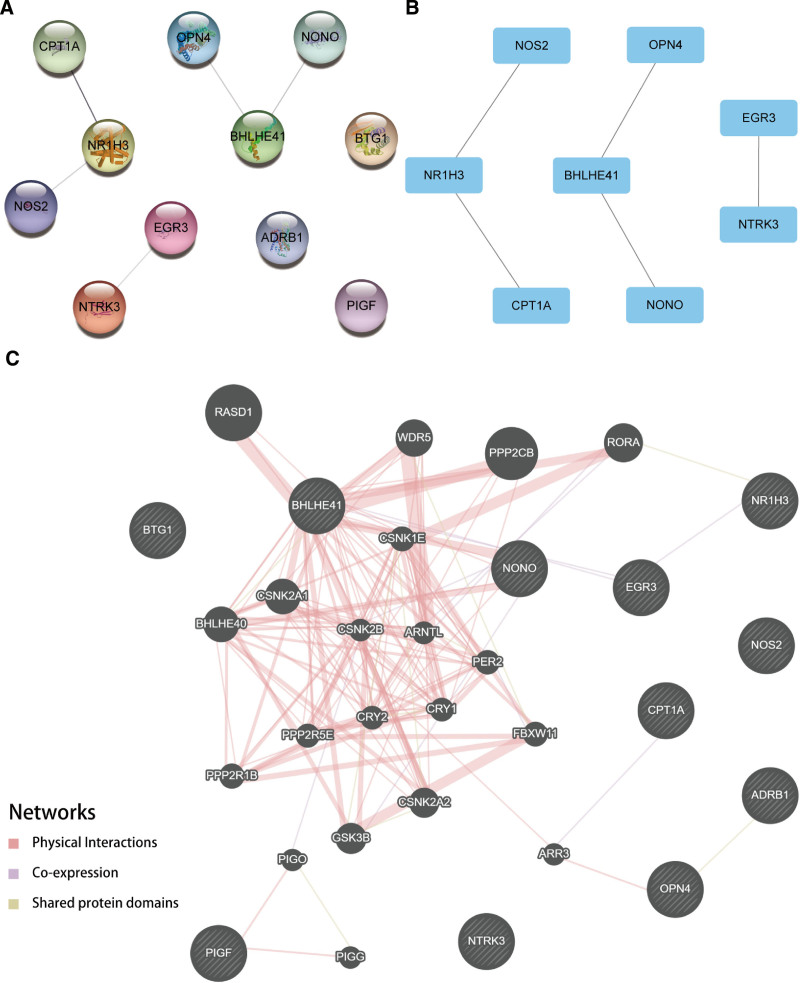
PPI interaction networks. (A) STRING visualizes the PPI Network for all genes. (B) Cytoscape visualizations of PPI networks for interacting genes only. (C) Interaction network of functionally similar genes of the GeneMANIA website predicting circadian rhythm prognosis-related genes. The Interstructured network of (A–B) was collected in the STRING database and constructed in Cytoscape software. The Interstructured network of C was collected at the GeneMANIA website and exported. The black circles with a white slash represent the input circadian rhythm prognosis-related genes, and other black circles without a white slash represent predicted functionally similar genes. The red lines represent genes with physical interactions, the purple lines represents co-expression relationships between genes, and the yellow lines represent having shared protein domains between genes. *P* < .05 was considered statistically significant. PPI = protein–protein interaction.

Eight PPI networks of prognostic CRRGs were constructed. The Cytoscape software was employed for interaction visualization (Fig. 9A and B). There are some interactions between NR1H3 and NOS2, NR1H3 and CPT1A, BHLHE41 and NONO, BHLHE41 and OPN4, and EGR3 and NTRK3 when the required minimum interaction score was 0.150. In addition, we also predicted and constructed the functional similar genes interaction network of the 11 prognostic CRRGs through the GeneMANIA website (Fig. 9C).

### 3.7. ROC curves

**Figure 10.**
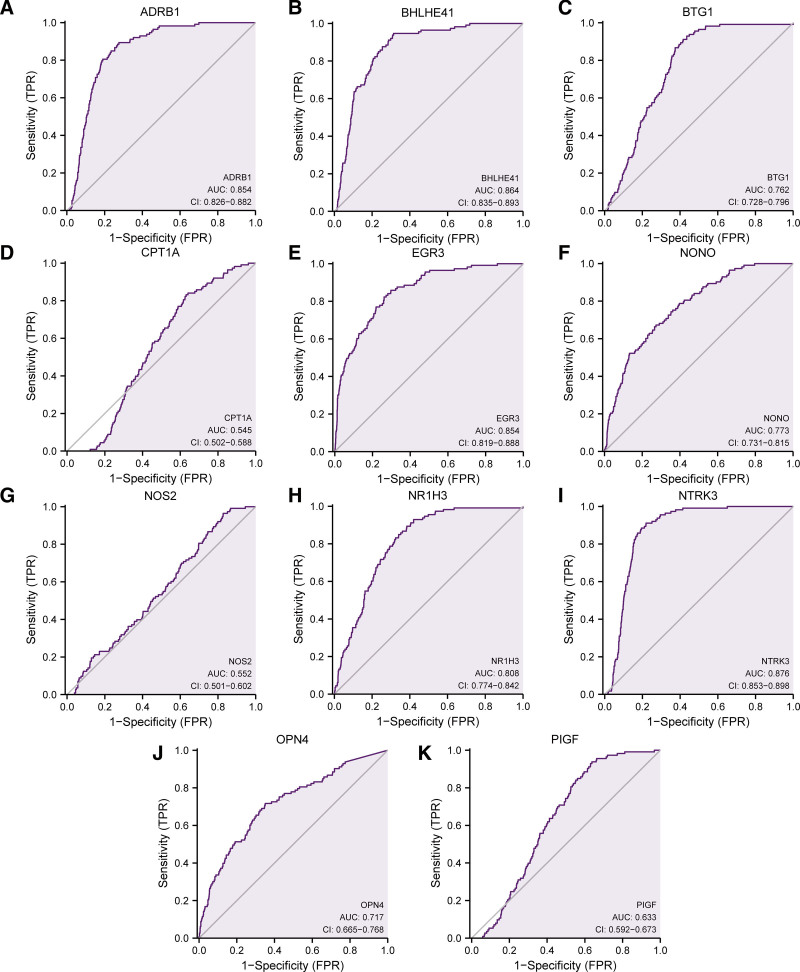
ROC curves. E.Results of ROC curves of the genes ADRB1 (A), BHLHE41 (B), BTG1 (C), CPT1A (D), EGR3 (E), NONO (F), NOS2 (G), NR1H3 (H), NTRK3 (I), OPN4 (J), PIGF (K) in TCGA-BRCA dataset. BRCA = breast cancer, ROC = receiver operating characteristic, TCGA = The Cancer Genome Atlas.

With the purpose of investigating the correlation of the expression of 11 prognostic CRRGs and the onset of BRCA, we made ROC curves of these 11 prognostic CRRGs (ADRB1, BHLHE41, BTG1, CPT1A, EGR3, NONO, NOS2, NR1H3, NTRK3, OPN4, PIGF) in TCGA-BRCA dataset, and the findings were displayed (Fig. 10A–E). As from the ROC curves in figure, the expression of ADRB1(AUC = 0.854, Fig. 10A), BHLHE41(AUC = 0.864, Fig. 10B), BTG1(AUC = 0.762, Fig. 10C), EGR3(AUC = 0.854, Fig. 10E), NONO (AUC = 0.773, Fig. 10F), NR1H3(AUC = 0.808, Fig. 10H), NTRK3(AUC = 0.876, Fig. 10I), OPN4(AUC = 0.717, Fig. 10J) showed a high correlation with the occurrence of BRCA. The correlation of the expression of CPT1A (AUC = 0.545, Fig. 10D), NOS2 (AUC = 0.552, Fig. 10G), PIGF (AUC = 0.633, Fig. 10K) and the occurrence of BRCA was general. We selected the genes (ADRB1, BHLHE41, BTG1, EGR3, NONO, NR1H3, NTRK3, OPN4, PIGF) (AUC > 0.6) as the key genes of BRCA.

### 3.8. Clinical correlation analysis of the prognosis

To further validate the established LAS prognostic model of LASSO regression, a statistical analysis was implemented (Table 6) on clinical information of BRCA patients obtained in TCGA-BRCA dataset. Afterward, an analysis was implemented on clinical correlation of the high and low prognosis expression of 9 prognostic CRRGs (ADRB1, BHLHE41, BTG1, EGR3, NONO, NR1H3, NTRK3, OPN4, PIGF) using univariate and multivariate BRCA dataset.

**Table 6:** Patient characteristics of BRCA patients in the TCGA datasets.

Characteristic	Levels	Overall
n		1083
T stage, n (%)	T1	277 (25.6%)
	T2	629 (58.2%)
	T3	139 (12.9%)
	T4	35 (3.2%)
N stage, n (%)	N0	514 (48.3%)
	N1	358 (33.6%)
	N2	116 (10.9%)
	N3	76 (7.1%)
M stage, n (%)	M0	902 (97.8%)
	M1	20 (2.2%)
Pathologic stage, n (%)	Stage I	181 (17.1%)
	Stage II	619 (58.4%)
	Stage III	242 (22.8%)
	Stage IV	18 (1.7%)
Age, n (%)	≤60	601 (55.5%)
	>60	482 (44.5%)
OS event, n (%)	Alive	931 (86%)
	Dead	152 (14%)
DSS event, n (%)	Alive	978 (92%)
	Dead	85 (8%)
PFI event, n (%)	Alive	936 (86.4%)
	Dead	147 (13.6%)
Age, median (IQR)		58 (48.5, 67)

BRCA = breast cancer, DSS = disease specific survival, IQR = interquartile range, OS = overall survival, PFI = progression free interval, TCGA = The Cancer Genome Atlas.

**Figure 11.**
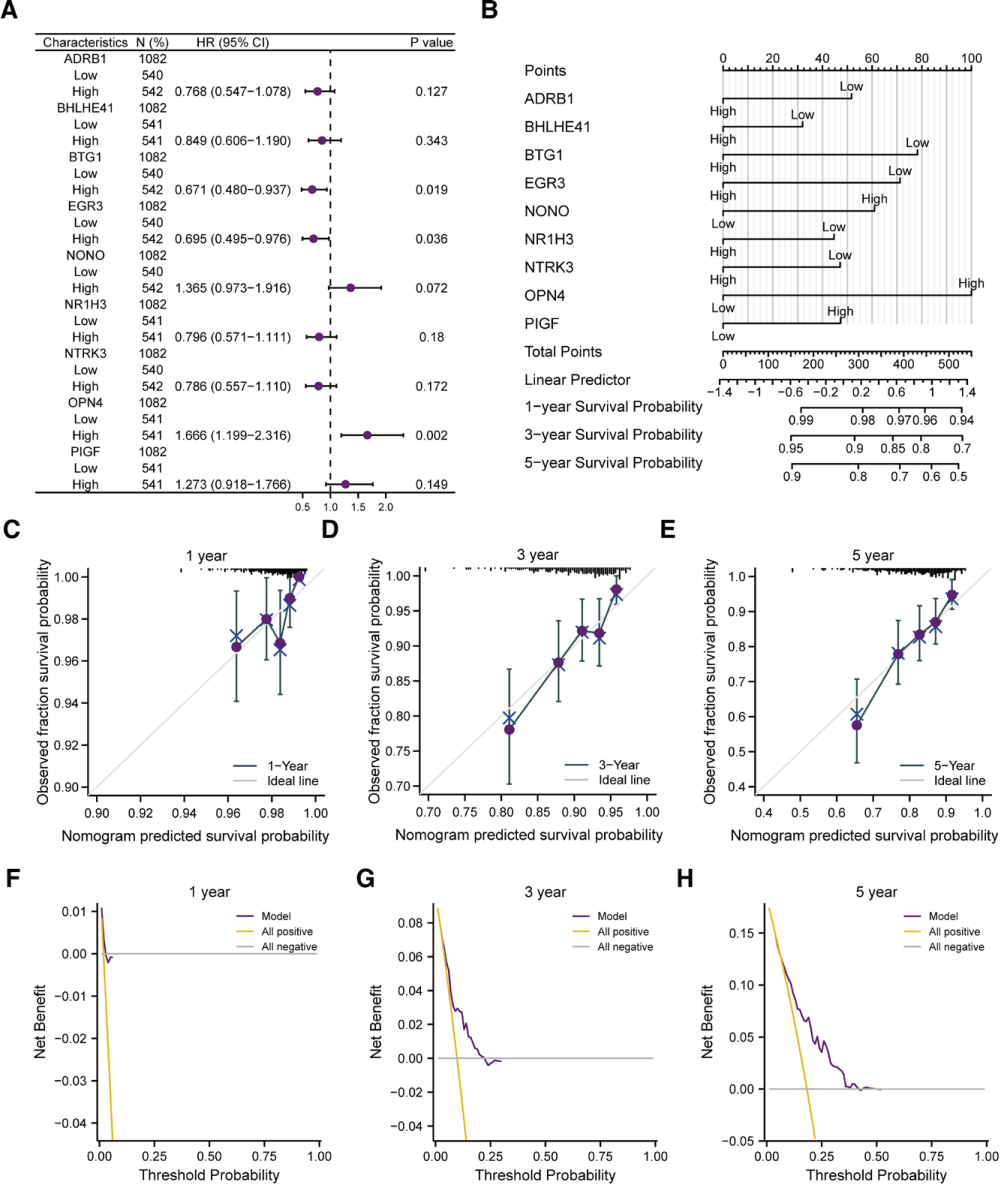
Prognostic correlation analysis. (B) Forest plot (A) and nomogram (B) for prognostic CRRGs of multivariate Cox regression analysis. (C–E) Calibration curve diagrams of 1-year (C), 3-year (D), 5-year (E) for nomogram analysis of univariate and multivariate Cox regression models. (F–H) DCA diagram of LASSO-Cox regression prognostic model for 1 year (F), 3 years (G), and 5 years (H). In DCA diagram, the x-axis corresponds to the threshold probability or probability threshold, while the y-axis denotes the net gain. CRRGs = circadian rhythm-related genes, DCA = decision curve analysis, LASSO = least absolute shrinkage and selection operator.

First, we implemented a univariate Cox regression analysis on prognostic CRRGs expression. Next, we established a multivariate Cox regression model (Table 7), incorporating factors with p-values <0.1 from the analysis. Forest plot was employed to display the outcomes (Fig. 11A). Next, to assess the model’s predictive ability, we implemented a nomogram-analysis on the genes selected in multivariate Cox regression model. Consequently, a nomogram was generated (Fig. 11B). In addition, we performed a Prognostic Calibration-analysis to nomogram (nomogram chart) at 1 C year (Fig. 11C), 3 years (Fig. 11D) and 5 years of the multivariate Cox regression model (Fig. 11E) and drew a calibration curve diagram (Fig. 11C–E). In the calibration curve, the purple line of 5 years is closest to the gray ideal line. It indicates that the model predicted better at year 5 than at 1 and 3 years. Subsequently We utilized the DCA analysis to evaluate the role of the LASSO-Cox regression prognostic model this study constructed in terms of clinical utility for 1 year (Fig. 11F), 3 years (Fig. 11G), and 5 years (Fig. 11H) and showed the results by Fig. 11E–H. The range of *x* values where the blue line of the representative model is stable above the red line of All positive and the gray line of All negative are the maximum at 5 years and 1 and 3 years are smaller. It indicates that the model predicted better at year 5 than at 1 and 3 years.

**Table 7:** Cox regression to identify hub genes and clinical features associated with OS.

Characteristics	Total (N)	Univariate analysis		Multivariate analysis	
		Hazard ratio (95% CI)	*P*-value	Hazard ratio (95% CI)	*P*-value
ADRB1	1082				
Low	540	Reference			
High	542	0.672 (0.487–0.926)	.015	0.768 (0.547–1.078)	.127
BHLHE41	1082				
Low	541	Reference			
High	541	0.685 (0.496–0.946)	.022	0.849 (0.606–1.190)	.343
BTG1	1082				
Low	540	Reference			
High	542	0.595 (0.432–0.821)	.002	0.671 (0.480–0.937)	.019
EGR3	1082				
Low	540	Reference			
High	542	0.566 (0.410–0.782)	<.001	0.695 (0.495–0.976)	.036
NONO	1082				
Low	540	Reference			
High	542	1.649 (1.191–2.285)	.003	1.365 (0.973–1.916)	.072
NR1H3	1082				
Low	541	Reference			
High	541	0.682 (0.493–0.944)	.021	0.796 (0.571–1.111)	.180
NTRK3	1082				
Low	540	Reference			
High	542	0.628 (0.456–0.865)	.004	0.786 (0.557–1.110)	.172
OPN4	1082				
Low	541	Reference			
High	541	1.409 (1.021–1.944)	.037	1.666 (1.199–2.316)	.002
PIGF	1082				
Low	541	Reference			
High	541	1.397 (1.013–1.927)	.041	1.273 (0.918–1.766)	.149

### 3.9. IHC analysis

**Figure 12.**
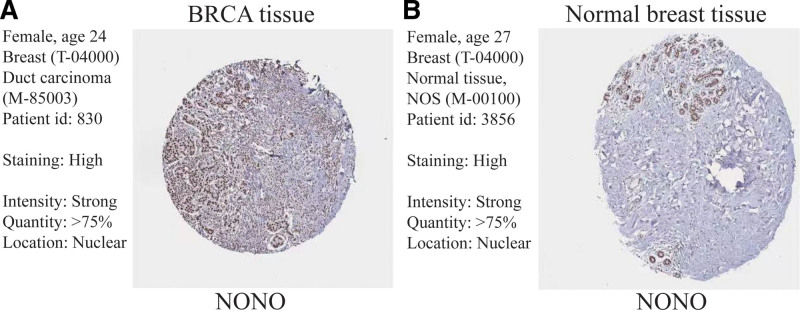
Immunohistochemical analysis. (A and B) Immunohistochemical analysis of the gene NONO in BRCA tissue (A) and Normal breast tissue (B). Data were obtained from the Human Protein Atlas database. BRCA = breast cancer.

An IHC analysis was implemented on gene expressions in BRCA tumor tissue and normal breast tissue, with HR values >1 (NONO, OPN4, PIGF) in the Cox model by using the Human Protein Atlas database. The stain were DAB (3,3-diaminobenzidine) and hematoxylin. Excluding genes not detected (OPN4, PIGF), gene NONO was more highly expressed in BRCA tumor tissue (BRCA tissue, Fig. 12A) than in normal breast tissue (Normal breast tissue, Fig. 12B).

## 4. Discussion

By 2020, BRCA had surpassed lung cancer to become the most prevalent cancer globally in terms of both incidence and mortality. BRCA has also emerged as the fifth leading cause of cancer-related deaths worldwide.^[27]^ The treatment of BRCA has made significant progress in personalized diagnosis and targeted therapy. For example, PARP inhibitors, such as olaparib and tarazoparib, have been widely used in neoadjuvant, adjuvant and advanced therapy. However, recurrence and metastasis are still the main challenges of current treatment, especially in the issue of drug resistance. In addition, the complexity and side effects of treatment methods also limit the treatment options for patients.^[28]^ Future studies may focus on the use of genomics and biomarkers to enhance the precision and efficacy of BRCA treatment, even to prevention of BRCA. It has been shown that clock genes could influence tumorigenesis.^[29]^ Clock genes play a direct role in controlling cell apoptosis or proliferation and cell division, cell cycle checkpoints, and response to DNA damage, thereby regulating gene expression and cell cycle.^[30]^ The alterations of these functions can increase the likelihood of cancer development. The exploration of molecular alterations in circadian rhythm-regulated genes within breast tumor cells remains largely uncharted. Therefore, identifying the molecular mechanisms underlying BRCA development is essential for advancing cancer therapy.

We established a prognostic model of CRRGs in BRCA patients to explore the relationship between the 2. Clock genes, constituting an auto-regulated transcription-translational loop of circadian genes, play a crucial part in driving circadian rhythms within individual cells.^[7]^ In contrast to neighboring normal cells in multiple tissues (i.e. endometrial cancer, breast tumors, lung cancer), circadian proteins undergo alterations in tumor cells.^[7]^ Studies have reported that some clock genes (i.e. Nr1d1, Npas2, Arntl2) might promote tumorigenesis, while others (i.e. Crys, Pers, Rors) might act as inhibitory tumorigenesis.^[31]^ However, it is not clear that whether the prognosis of BRCA patients are associated with CRRGs.

Gene-expression, corresponding clinical profiles and samples data of BRCA patients was acquired from GEO, UCSC Xena and TCGA. And comprehensive information about the human gene set was acquired from MSigDB. We selected 282 CRRGs in total from a literature of the PubMed website by organizing, merging, and eliminating the weight. Then, a prognostic multigene signature incorporating circadian rhythm-related DEGs was constructed, and the predictive power was validated. GEO database was employed to validate the credibility of the prognostic multigene signature. Finally, FEA was conducted to explore association between BP, MF, CC and BRCA.

In our research, the prognostic model constructed consisted of 20 CRRGs (ADORA1, ADRB1, BHLHE41, BTG1, CPT1A, EGR3, GHRHR, NCOA2, NLGN1, NONO, NOS2, NR1H3, NTRK3, OPN4, PIGF, PSPC1, RELB, SIAH2, SMARCD3, WDR5). In this study, the gene expression of ADORA1 exhibited a significant difference between HRG and LRG. Although the role of ADRB1 in breast cancer has not been fully studied, its role in other tumors suggests that it may also play an important role in breast cancer. As a basic helix-loop-coil transcription factor, BHLHE41, responsible for inhibiting E-box-mediated transcription, underlies a negative feedback loop in circadian clock. Carriers of the Tyr362His (Y362H) variant of the BHLHE41 gene have been reported to have shorter sleep duration and are resistant to sleep deprivation. NONO/nrb54 refers to an octamer-binding protein that does not contain a POU domain and has multiple MF in gastric cancer, lung cancer, and prostatic cancer.^[32]^ The analysis in our research demonstrated that NONO expression is a high correlation with the occurrence of BRCA as previously reported.^[33,34]^ NONO regulates the proliferation and metastasis of breast cancer cells by affecting the expression of multiple genes. For example, it has been found that NONO can regulate the proliferation of breast cancer cells by affecting the expression of SKP2 and E2F8. In addition, NONO also interacts with nuclear PKM2 to activate SERPINE1 transcription by regulating histone H3 phosphorylation and acetylation, thereby promoting metastasis of triple-negative breast cancer.^[35]^ These studies suggest that NONO plays an important role in the malignant progression of breast cancer and may be a potential target for breast cancer therapy.^[36]^ WDR5 is renowned for its pivotal function within the KMT2 complexes, promoting transcriptional processes via the methylation of H3K4.^[37]^ Knocking down, tumorigenicity and metastatic capabilities of BRCA cells was impaired independently.^[37,38]^ The functional roles of EGR3, ADRB1, OPN4, NLGN1 in distinct aspects of BRCA progression remains unclear. Although earlier researches have pointed out that these genes is extremely critical in the pathogenesis of BRAC, combining the 2 as a prognosis indicator for patients with BRCA is the first time by us.

We performed a GO gene FEA for these 20 prognostic CRRGs. The results showed that these 20 prognostic CRRGs were mainly enriched in circadian rhythm in BRCA, regulation of circadian rhythm, circadian behavior. This provides support for some recent studies showing that onset and progression of BRCA are affected by circadian rhythms.^[39,40]^ Circadian rhythm disorders, stemming from shift work and evening light exposure, contribute to an elevation of the BRCA incidence by 19% and 12%, accordingly.^[7]^ Based upon the findings, the 20 genes exhibited enrichment in other BP, and nuclear matrix, nuclear periphery, integral component of synaptic membrane and other CC, and the E-box binding, enhancer sequence-specific DNA binding, enhancer binding, and other MF. The result is not surprising. Previous studies have found that clock genes regulate homeostasis of the body through the modulation of cellular metabolism, proliferation, and diverse gene expression pathways. Circadian rhythm is formed by clock gene oscillations at the molecular level.^[41]^

Despite several positive findings in this research, there are still some limitations. As a retrospective study, data loss and selection bias are inevitably. Besides, the prognostic model relies upon a publicly accessible repository. Although this study has validated the prognostic model using independent datasets (GSE42568 and GSE102484) from the GEO database, and 10-fold cross-validation and LASSO methods were used to enhance the robustness of the model, we realize that further additional validation using external datasets (such as METABRIC) would be more comprehensive. In addition, experimental verification at the protein level, such as Western blot, helps to further confirm the role of key genes. In the future, more external data sets and cell experiments should be further introduced to enhance the reliability of the study. While exhibiting commendable performance in the analysis, the predictive efficacy necessitates further validation through rigorous randomized controlled trials. Furthermore, this study is less informative about causality.

## 5. Conclusions

In summary, the research built a novel prognostic model of 20 CRRGs, and its robust prediction efficacy was validated. And we found some correlation between some gene expression and the occurrence of BRCA, which might be helpful for exploring the occurrence of BRCA and play a guiding role in improving BRCA.

## Acknowledgments

The authors thank the TCGA and GEO research networks for providing open-access data. No individuals requiring Acknowledgments by name were included in this study.

## Author contributions

**Conceptualization:** Jian Wu, Hong Liu, Jiechao Ren.

**Data curation:** Jian Wu.

**Formal analysis:** Jian Wu.

**Funding acquisition:** Tielin Wang.

**Investigation:** Tielin Wang, Ying Liu.

**Methodology:** Tielin Wang, Ying Liu.

**Project administration:** Ying Liu.

**Software:** Yan Zhang.

**Supervision:** Yan Zhang.

**Validation:** Jiechao Ren.

**Visualization:** Jiechao Ren.

**Writing – original draft:** Tielin Wang, Hong Liu.

**Writing – review & editing:** Tielin Wang.

## Supplementary Material



## References

[1] Han L Yan Y Zhao L. LncRNA HOTTIP facilitates the stemness of breast cancer via regulation of miR-148a-3p/WNT1 pathway. J Cell Mol Med. 2020;24:6242–52. 32307830 10.1111/jcmm.15261 PMC7294123

[2] Zuo S Yu J Pan H Lu L. Novel insights on targeting ferroptosis in cancer therapy. Biomark Res. 2020;8:50. 33024562 10.1186/s40364-020-00229-w PMC7532638

[3] Zhang D Yuan Y Xiong J. Anti-breast cancer effects of dairy protein active peptides, dairy products, and dairy protein-based nanoparticles. Front Pharmacol. 2024;15:1486264. 39605907 10.3389/fphar.2024.1486264 PMC11598434

[4] Zhang D Yuan Y Zeng Q. Plant protein-derived anti-breast cancer peptides: sources, therapeutic approaches, mechanisms, and nanoparticle design. Front Pharmacol. 2025;15:1468977. 39898323 10.3389/fphar.2024.1468977 PMC11783187

[5] Xiong X Zheng LW Ding Y. Breast cancer: pathogenesis and treatments. Sig Transduct Target Ther. 2025;10:1–33. 10.1038/s41392-024-02108-4 PMC11836418 39966355

[6] Zeng Y Guo Z Wu M Chen F Chen L. Circadian rhythm regulates the function of immune cells and participates in the development of tumors. Cell Death Discov. 2024;10:1–17. 38678017 10.1038/s41420-024-01960-1 PMC11055927

[7] Zhou Z Zhang R Zhang Y. Circadian disruption in cancer hallmarks: Novel insight into the molecular mechanisms of tumorigenesis and cancer treatment. Cancer Lett. 2024;604:217273. 39306230 10.1016/j.canlet.2024.217273

[8] Rashed N Liu W Zhou X Bode AM Luo X. The role of circadian gene CLOCK in cancer. Biochim Biophys Acta. 2024;1871:119782. 10.1016/j.bbamcr.2024.119782 38871225

[9] Ze Y Wu Y Tan Z. Signaling pathway mechanisms of circadian clock gene Bmal1 regulating bone and cartilage metabolism: a review. Bone Res. 2025;13:19. 39870641 10.1038/s41413-025-00403-6 PMC11772753

[10] El-Tanani M Rabbani SA Ali AA. Circadian rhythms and cancer: implications for timing in therapy. Discov Oncol. 2024;15:767. 39692981 10.1007/s12672-024-01643-4 PMC11655929

[11] Travaglino A Raffone A Mascolo M. TCGA molecular subgroups in endometrial undifferentiated/dedifferentiated carcinoma. Pathol Oncol Res. 2020;26:1411–6. 31811476 10.1007/s12253-019-00784-0

[12] Xu M Zhou H Hu P. Identification and validation of immune and oxidative stress-related diagnostic markers for diabetic nephropathy by WGCNA and machine learning. Front Immunol. 2023;14:1084531. 36911691 10.3389/fimmu.2023.1084531 PMC9992203

[13] Ahl H Lajr C Vad S. Exploring the potential of olfactory receptor circulating RNA measurement for preeclampsia prediction and its linkage to mild gestational hypothyroidism. Int J Mol Sci. 2023;24:24. 10.3390/ijms242316681 PMC10706743 38069004

[14] Zeng X Shi G He Q Zhu P. Screening and predicted value of potential biomarkers for breast cancer using bioinformatics analysis. Sci Rep. 2021;11:20799. 34675265 10.1038/s41598-021-00268-9 PMC8531389

[15] Fang Y Zhang Q Guo C. Mitochondrial-related genes as prognostic and metastatic markers in breast cancer: insights from comprehensive analysis and clinical models. Front Immunol. 2024;15:1461489. 39380996 10.3389/fimmu.2024.1461489 PMC11458410

[16] Yi X Li J Zheng X. Construction of PANoptosis signature: novel target discovery for prostate cancer immunotherapy. Mol Ther Nucleic Acids. 2023;33:376–90. 37547288 10.1016/j.omtn.2023.07.010 PMC10400972

[17] Zhou R Chen X Liang J. A circadian rhythm-related gene signature associated with tumor immunity, cisplatin efficacy, and prognosis in bladder cancer. Aging (Milano). 2021;13:25153–79. 10.18632/aging.203733 PMC8714136 34862329

[18] Liu Y Zhao Y Zhang S. Developing a prognosis and chemotherapy evaluating model for colon adenocarcinoma based on mitotic catastrophe-related genes. Sci Rep. 2024;14 :1655. 38238555 10.1038/s41598-024-51918-7 PMC10796338

[19] Yang L Yu X Liu M Cao Y. A comprehensive analysis of biomarkers associated with synovitis and chondrocyte apoptosis in osteoarthritis. Front Immunol. 2023;14:1149686. 37545537 10.3389/fimmu.2023.1149686 PMC10401591

[20] Wu T Hu E Xu S. clusterProfiler 4.0: a universal enrichment tool for interpreting omics data. Innovation (Camb). 2021;2:100141. 34557778 10.1016/j.xinn.2021.100141 PMC8454663

[21] Wu B Xi S. Bioinformatics analysis of differentially expressed genes and pathways in the development of cervical cancer. BMC Cancer. 2021;21: 733. 34174849 10.1186/s12885-021-08412-4 PMC8236200

[22] Szklarczyk D Kirsch R Koutrouli M. The STRING database in 2023: protein-protein association networks and functional enrichment analyses for any sequenced genome of interest. Nucleic Acids Res. 2023;51:D638–46. 36370105 10.1093/nar/gkac1000 PMC9825434

[23] Shannon P Markiel A Ozier O. Cytoscape: a software environment for integrated models of biomolecular interaction networks. Genome Res. 2003;13:2498–504. 14597658 10.1101/gr.1239303 PMC403769

[24] Senguttuvan NB Subramanian V Venkatesan V Muralidharan TR Sankaranarayanan K. Clonal hematopoiesis of indeterminate potential (CHIP) and cardiovascular diseases-an updated systematic review. J Genet Eng Biotechnol. 2021;19:105. 34279740 10.1186/s43141-021-00205-3 PMC8287286

[25] Nahm FS. Receiver operating characteristic curve: overview and practical use for clinicians. Korean J Anesthesiol. 2022;75:25–36. 35124947 10.4097/kja.21209 PMC8831439

[26] Engebretsen S Bohlin J. Statistical predictions with glmnet. Clin Epigenetics. 2019;11:123. 31443682 10.1186/s13148-019-0730-1 PMC6708235

[27] Sung H Ferlay J Siegel RL. Global Cancer Statistics 2020: GLOBOCAN estimates of incidence and mortality worldwide for 36 cancers in 185 countries. CA Cancer J Clin. 2021;71:209–49. 33538338 10.3322/caac.21660

[28] Arun B Couch FJ Abraham J Tung N Fasching PA. BRCA-mutated breast cancer: the unmet need, challenges and therapeutic benefits of genetic testing. Br J Cancer. 2024;131:1400–14. 39215191 10.1038/s41416-024-02827-z PMC11519381

[29] Li M Cai Y Zhang M Deng S Wang L. NNBGWO-BRCA marker: neural network and binary grey wolf optimization based Breast cancer biomarker discovery framework using multi-omics dataset. Comput Methods Programs Biomed. 2024;254:108291. 38909399 10.1016/j.cmpb.2024.108291

[30] Dubsky P Jackisch C Im SA. BRCA genetic testing and counseling in breast cancer: how do we meet our patients’ needs? NPJ Breast Cancer. 2024;10:1–12. 39237557 10.1038/s41523-024-00686-8 PMC11377442

[31] Wang MH Liu X Wang Q Zhang HW. A circadian rhythm-related gene signature for prognosis, invasion and immune microenvironment of breast cancer. Front Genet. 2023;13:1104338. 36685904 10.3389/fgene.2022.1104338 PMC9849377

[32] Bret C Desmots-Loyer F Moreaux J Fest T. BHLHE41, a transcriptional repressor involved in physiological processes and tumor development. Cell Oncol. 2025;48:43–66. 10.1007/s13402-024-00973-3 PMC11850569 39254779

[33] Kim SJ Ju JS Kang MH. RNA-binding protein NONO contributes to cancer cell growth and confers drug resistance as a theranostic target in TNBC. Theranostics. 2020;10:7974–92. 32724453 10.7150/thno.45037 PMC7381744

[34] Iino K Mitobe Y Ikeda K. RNA-binding protein NONO promotes breast cancer proliferation by post-transcriptional regulation of SKP2 and E2F8. Cancer Sci. 2020;111:148–59. 31733123 10.1111/cas.14240 PMC6942431

[35] Zhu Z Zhao X Zhao L. p54(nrb)/NONO regulates lipid metabolism and breast cancer growth through SREBP-1A. Oncogene. 2016;35:1399–410. 26148231 10.1038/onc.2015.197

[36] Lone BA Siraj F Sharma I. Non-POU domain-containing octomer-binding (NONO) protein expression and stability promotes the tumorigenicity and activation of Akt/MAPK/β-catenin pathways in human breast cancer cells. Cell Commun Signal. 2023;21:157. 37370134 10.1186/s12964-023-01179-0 PMC10294335

[37] Mitchell K Sprowls SA Arora S. WDR5 represents a therapeutically exploitable target for cancer stem cells in glioblastoma. Genes Dev. 2023;37:86–102. 36732025 10.1101/gad.349803.122 PMC10069451

[38] Cai WL Chen JFY Chen H. Human WDR5 promotes breast cancer growth and metastasis via KMT2-independent translation regulation. Elife. 2022;11:e78163. 36043466 10.7554/eLife.78163 PMC9584608

[39] Chi L Zou Y Qin L. TIMELESS contributes to the progression of breast cancer through activation of MYC. Breast Cancer Res. 2017;19:53. 28464854 10.1186/s13058-017-0838-1 PMC5414141

[40] Angelousi A Kassi E Ansari-Nasiri N Randeva H Kaltsas G Chrousos G. Clock genes and cancer development in particular in endocrine tissues. Endocr Relat Cancer. 2019;26:R305–17. 30959483 10.1530/ERC-19-0094

[41] Shi T Min M Sun C Zhang Y Liang M Sun Y. Does insomnia predict a high risk of cancer? A systematic review and meta-analysis of cohort studies. J Sleep Res. 2020;29:e12876. 31352687 10.1111/jsr.12876

